# Prospects for commercial production of diatoms

**DOI:** 10.1186/s13068-017-0699-y

**Published:** 2017-01-18

**Authors:** Jaw-Kai Wang, Michael Seibert

**Affiliations:** 1 Shenzhen Jawkai Bioengineering R&D Center, Inc., Building #9, Marine Biotech Industrial Park, Dapeng District, Shenzhen, 518120 China; 2 National Renewable Energy Laboratory, BioEnergy Science and Technology Directorate, Golden, CO 80401 USA

**Keywords:** Diatoms, Biomass, Long-term growth outdoors, Invasive species, Biofuels, Bioproducts, Co-products, Hydrothermal liquefaction

## Abstract

In this review, a simple procedure that portends the open-pond growth of commercially viable diatoms is discussed. We examined a number of topics relevant to the production and harvesting of diatoms as well as topics concerning the production of bioproducts from diatoms. Among the former topics, we show that it is currently possible to continuously grow diatoms and control the presence of invasive species without chemical toxins at an average annual yield of 132 MT dry diatoms ha^−1^ over a period of almost 5 years, while maintaining the dominancy of the optimal diatom species on a seasonal basis. The dominant species varies during the year. The production of microalgae is essentially agriculture, but without the ability to control invasive species in the absence of herbicides and insecticides, pollution and production costs would be prohibitive. Among the latter topics are the discussions of whether it is better to produce lipids and then convert them to biofuels or maximize the production of diatom biomass and then convert it to biocrude products using, for example, hydrothermal processes. It is becoming increasingly evident that without massive public support, the commercial production of microalgal biofuels alone will remain elusive. While economically competitive production of biofuels from diatoms will be difficult, when priority is given to multiple high-value products, including wastewater treatment, and when biofuels are considered co-products in a systems approach to commercial production of diatoms, an economically competitive process will become more likely.

## Background

Diatoms are very promising microorganisms for biofuels production [1], because (a) their ubiquitous presence and competitive advantage up against other microalgae (under suitable, controllable conditions) will allow for continuously varying the species that is cultivated to follow seasonal variations in the available optimal organisms; (b) they grow rapidly, doubling their biomass in a few hours; (c) their growth can be easily controlled by the availability of silicate; and (d) almost all of their biomass can be put to profitable use. Among the advantages of a diatom-based, open (to the atmosphere) pond system in biofuels production are the simultaneous capability of assimilating carbon dioxide and removing nutrients from wastewater sources, while at the same time, producing valuable fuels and other bioproducts.

An economically competitive, open-diatom production system is must among many challenges solve two significant problems: (a) prevention of invasive species from becoming the dominant species, thus hindering the operator’s ability to ensure dominance of the most desirable species in a production facility and (b) control of damage by predators when they do invade. In agriculture, these tasks are often handled by herbicide and pesticide treatments. However, in the cultivation of aquatic plants, including diatoms, toxic chemicals cannot be used for environmental reasons and because in controlling destructive species and other undesirable microorganisms, these toxins can also eliminate the very aquatic organisms that are wanted.

## Diatoms

Diatoms are photosynthetic, eukaryotic microalgae not only found, for example, in the *Bacillariophyta* family with which we work, but they are also found in other families [2]. There are more than 200 genera of extant diatoms and approximately 100,000 living species [2, 3]. Diatoms contain a wide variety of lipids, including membrane-bound polar lipids, triglycerides, and free fatty acids [4, 5]. Compounds such as sterols, waxes, and acyl lipids have also been identified. Increased lipid concentrations within different species of diatoms have been observed by the modification of nutrient availability and other requisite growth conditions [6–11]. Syvertsen [10] has concluded that to maximize diatom fatty acid production, it is best to maximize total diatom production. Notably, lipid fractions as high as 70–85% have been reported in some diatoms [12], but 15–25% is more typical. High growth rates combined with significant lipid productivities make diatoms a leading candidate as a source of either bio-oil or biocrude. Bio-oil refers to the oil extracted from diatom lipid that can be upgraded using processes such as transesterification, and biocrude refers to the natural crude-like oil converted from the diatom biomass via thermochemical means. However, this latter opportunity has not been explored fully at this point of time [13, 14].

The dependence on silicates by diatoms is probably the key to their competitive success relative to other microalgae in our growth studies, though the ability of diatoms to sequester other nutrients and a more advanced carbon flux metabolism compared to other microalgae also contribute to their success [15]. Egge and Aksnes [16] stated that “diatoms use silicate as a regulating nutrient, which enhances phytoplankton competition in the ocean.” They also reported that diatom dominance in the ocean occurs regardless of the time of year, if a threshold concentration of 2 µM silicates is exceeded, and when this occurs diatoms nominally represent greater than 70% of the phytoplankton found in the ecosystem. In silicified plants, Raven [17] noted that, “relative to organic cell walls, biosilicate cell walls require less energy to synthesize (approximately 8% that of a comparable organic cell wall), representing a significant saving in the overall cell energy budget.” The same should be true in the case of diatom frustules. Other investigators have hypothesized that the frustule biosilicate [18] (in silicified cell wall of diatoms) acts as a pH buffering material [19], which facilitates shifting of bicarbonate to CO_2_ dissolved in cell fluids (the latter is readily metabolized by diatoms). Notwithstanding any other advantages imposed by silicate, diatoms under natural conditions in the ocean outcompete other algae of similar size due to superior growth rates [20].

## Mitigation of carbon dioxide and other pollutants

The ability of an open, microalgal-production facility to absorb CO_2_ (along with some pollutants simultaneously) can be very useful in symbiotic power generation/diatom production, oil refining/diatom production, or brewery/diatom production relationships. Besides CO_2_, some microalgae cultures, including diatoms can remove biological NOx from combustion gases [21, 22]. Similarly, the ability to remove nutrients can also be used to advantage in the treatment of wastewater.

The ability to produce biocrude, while consuming carbon dioxide and using only wastewater or water sources that are unsuitable for human consumption and irrigation, as well as using non-productive land, offers countries such as China and the United States real hope within the foreseeable future for achieving, to some degree, self-sufficiency in sustainable liquid biofuel production (however, recent improvements in fossil-fuel extraction technologies in the US may affect the timeline for implementing diatom, biocrude technologies).

It is also important to note that diatoms, when used in liquid fuel production, will leave very little waste material (pollutants) behind. The residual growth medium can be recycled, with much organic carbon in the diatoms converted into biocrude (with thermal processing), and nutrients remaining in the post-processed water reused. Furthermore, proteins produced (with non-thermal processing) are excellent animal feed, and the frustules, which are covered with nano-sized holes, can be used to remove heavy metals from industrial wastewater. Our own experience in China indicates that, for example, up to 99.9% of the copper can be removed from industrial wastewater by filtering the water through diatom frustules [23].

## Production and harvesting

**Fig. 1:**
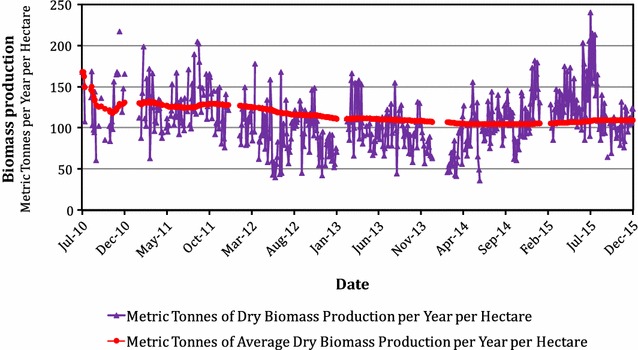
Diatom yields in Shenzhen, China from July 1, 2010 to December 1, 2015 for the open, 180 l photobioreactor system described in the text. Note that the species of diatom changes with time of year

Although basic photosynthetic mechanisms in diatoms and other microalgae are similar to those found in higher plants, microalgae can produce as much as 30 times the volume of oil per unit land area, compared to commercial oilseed crops [24]. The reason is that single-cell organisms are more efficient solar energy converters because they do not need to support an array of non-photosynthetic cells as in plants. Furthermore, because they grow in an aqueous environment, they are inherently more efficient at accessing water, CO_2_ (using a carbon-concentrating mechanism), and dissolved nutrients. Weyer et al. [25] calculated the theoretical maximum efficiency for algal production at 354,000 L per hectare per year of bio-oil, while the best case for real world production should be 40,700–53,000 L per hectare per year of bio-oil [25]. Against the best real world production estimates made by Weyer et al., we should take note (as shown later in Fig. 1) that in Shenzhen, China, the Jawkai Bioengineering R&D Center has already achieved a sustained diatom yields of over 120 MT of dry weight per hectare per year. Using the HTL process (see the section on "Hydrothermal liquefaction" below), one-third of the diatom dry weight can be converted to biocrude and that is over 36,000 L of biocrude per hectare (calculated based on a 300-day per year production basis; data not shown).

Marine diatoms might also, in fact, be cultivated in the open ocean [26]. Historically, off-shore aquaculture of kelp was investigated in the 1970s [27] when the U.S. Navy brought engineers, oceanographers, and marine biologists together to explore (a) the feasibility of designing and deploying underwater structures that could withstand storms, (b) ways of dealing with the logistics of working many kilometers from land, and (c) issues in determining if there were sufficiently high returns to offset the costs. Similar approaches to producing diatoms in the ocean would have a number of advantages, including overcoming the heat removal challenge and a major problem in closed production systems. A closed production system floating on the water can dissipate heat at very little cost. Recently, NASA has investigated the technical feasibility of a unique floating algae-cultivation system (http://www.nasa.gov/centers/ames/research/OMEGA/). The research has demonstrated that their OMEGA system is effective in growing microalgae and treating wastewater on a small scale, but the economic feasibility of this scheme remains to be shown.

### Open system production

Aquaculturists have traditionally initiated diatom production in closed, batch systems before seeding them into open ponds [28]. However, large scale, closed, continuous production systems have many difficulties. The main ones are initial capital costs, the need to remove heat, and the overwhelming difficulty and high cost of maintenance activities. Fortunately, marine diatoms such as *Chaetoceros* sp. have been continuously cultivated successfully in a commercial, open-production system in Hawaii. For example, the Kona Bay Oyster and Shrimp Company in Kona, Hawaii, has successfully operated a commercial open system since the late 1990s, producing the marine diatom, *Chaetoceros*, to feed shrimp brood stock and bivalves. Basically, if one can maintain *Chaetoceros*, or any other diatom, in an open system, growing at log phase by controlling, among other things the concentration of silicate, then it will outgrow non-diatom species and thereby maintain its dominant position in the production system [29]. The Kona system was designed to produce relatively low density diatoms for shrimp production. However, the basic pilot plant has been further developed in China for high yield per unit surface area, and a patent was issued to Wang [30]. The control of aquatic animals that feed on diatoms has also been achieved in this system, and additional patents have been filed [31, 32]. Basically, the control of predators can be achieved by taking cognizance of the fact that plants are more resistant to adverse circumstance than animals and that a fast growing, single-cell organism (e.g., *Chaetoceros*) can recover from adverse conditions much quicker than aquatic animals. By repeatedly applying stresses (e.g., pH stress), the impact of predators can be minimized.

### Diatom yields

If photobioreactors are used, they need to be inexpensive in order to meet the stringent cost limitations required by the biofuel production industry. Unfortunately, any photobioreactor that is made up of transparent material of sufficient strength is likely to be too expensive. A high-yielding photobioreactor should have a cell density of at least 1 g/L at the peak. At an average weight of 4 pg per individual diatom cell, such as in *Chaetoceros*, the required cell density range would be between 5 and 10 × 10^6^ cells per ml. At this cell density, effective solar penetration starts to become limiting with depth, and it is wise to keep the effective penetration depth to within 30 cm. To utilize the incoming solar energy effectively, it is also desirable to have the diatom cells well mixed in the growth medium. Diatoms are known to be less sensitive to ocean turbulence than other phytoplankton [33]. However, while there is general agreement that turbulence is beneficial for the overall production of microalgae, no study has been done to quantify the effects of turbulence on the production rate of diatoms.

Figure 1 shows the yield of diatoms in Shenzhen, China, from 1 July, 2010, to 1 December, 2015. The growth tank units were clear plexiglass cylinders, 0.5 m in diameter by 1 m in height, with the top open to the atmosphere. This was a test system to examine long-term production yields, but a commercial system would have to use much larger, open ponds for cost reasons. Each unit was fed with 1 L of fine air bubbles per minute to stir the medium in the reactor. Forty vessels were placed one meter apart (center to center) and filled with 180 L of growth medium each. Daily harvesting was done at about 6 pm by centrifugation. The concentrated diatom paste was then freeze-dried for dry weight determinations. Depending upon the climate conditions, about 20% of the production was left behind each day to re-seed the tank units. The data show an average daily yield of 0.44 MT of dry diatoms per hectare per day. Using 300 useful days per year (February and March are normally not the best months for the production of diatoms and were not used in Fig. 1), the average annual yield was close to 132 MT of dry diatoms per hectare of active production surface area (the geometry was defined above). We recognize that this is a very high yield. For example, Huntley et al. [14] reported a yield of 75 MT ha^−1^ year^−1^ (but projected 100 MT ha^−1^ year^−1^) for the diatom, *Staurosira*, or 56% of the yield that we observe with *Chaetoceros* sp. To achieve this high yield, our production process included the following: (a) semi-continuous harvesting (done once a day), leading to optimal light absorption throughout the year; (b) optimal strains grown throughout the year for the Shenzhen coastal area (the changing dominant strain was obtained from nearshore ocean water, which continuously seeded the test bioreactors; thus, we always used the most productive local strain for the local conditions at all times during the year); (c) substantial (but not as yet perfect) control of invasive strains; (d) sufficient control of aquatic animals that feed on diatoms; (e) optimal temperature at all times (with ocean water as the coolant when necessary); (f) optimal nutritional supplementation present at all times; and (g) diatoms mostly in the log phase of their life cycle over extended periods of time (see next section). Using all of these techniques, we achieved higher production rates than possible with batch cultures under identical climate conditions.

We emphasize that our yields break none of the laws of thermodynamics, and in fact, our yields are less than half the 330–420 MT ha^−1^ year^−1^ that Weyer et al. [25] (see their Table 1) reported for their projected best case algal daily growth rate. Furthermore, this reference gives an even higher value for the theoretical limit. At this growth rate, the lipid content of the diatom dry weight varied between 15 and 20%, but using a HTL (see the section below on HTL for more details) conversion process in short term, lab-scale experiments, actual biocrude yields ranged between 35 and 50% of volatile solids (on a dry matter basis; data from Zhang on our diatom biomass [as shown later in the tables]) were obtained.

### Optimization of growth efficiency

It is important to note that most bio-production processes, such as farming, are essentially batch processes. For example after planting corn, the farmer waits for months and then harvests at maturity. At the beginning, the corn plant does not take full advantage of the resources (particularly solar radiation and land) available. The longer the time required for plant growth, the less efficient is the plant’s ability to utilize the total available solar energy and land area during its life cycle. A diatom like *Chaetoceros* has a very short growth cycle [34]. Under sub-tropical conditions as mentioned above, Wang and his group have demonstrated (unpublished work) that using 20% of the diatoms produced during the previous day as seed inoculum, their growth test systems (see Fig. 1) fully restored the previous diatom density during the following day. Nevertheless, this is still a semi-continuous batch process, repeated on a daily basis. Since we now have the capability of continuously harvesting *Chaetoceros*, or other diatoms, by foam fractionation (see next section), we can potentially maintain the algal density at close to optimal levels at all times, thus further increasing the potential ability of the culture to convert solar energy, CO_2_, and water to cell biomass.

If one studies Fig. 1 carefully, a high day-to-day variation of the yield data is apparent. The climate in Shenzhen is sub-tropical and cannot explain this variation. We suspect that the variation was caused by instability in our CO_2_ (CO_2_-saturated seawater) injection system (which was physically separate from our air-stirring system). Alternatively, and more likely, it might have been caused by intermittent over saturation of O_2_ from the stirring system, which would limit diatom growth. Production system control problems will be studied in future research.

### Harvesting

The separation of microalgae from used growth medium can be energy intensive. Traditional methods, such as centrifuging, filtering, or flocculation, are either energy intensive, difficult to operate, or require the introduction of chemicals into the process. In the latter case, chemicals might have to be removed before the diatoms could be used as a source of biocrude or animal feed. Naturally occurring surfactants, which are produced by the microalgae themselves (such as in the case with *Chaetoceros*), could provide a partial solution. Such surfactants would allow the use of foam fractionation to concentrate diatoms, and the technique was first demonstrated by Yuan [35], working under Wang. The concept was further developed by Csordas and Wang [36], who showed that foam fractionation can remove up to 90% of the *Chaetoceros* from its culture medium (the medium can then be reused with suitable processing after the diatoms are removed).

The ability to harvest diatoms efficiently and inexpensively is extremely crucial for maximizing diatom yield. We are harvesting once a day in Hawaii and China, but as mentioned in the last section, it can be argued that more frequent harvesting can increase the yield. Ideally, continuous harvesting in the future should give the best yields.

## Biofuels from diatoms

Obtaining biofuels from diatoms, or any microalga, could be achieved via two options: direct extraction of lipid and then processing into biofuel, or thermochemical conversion of the entire biomass fraction into a biocrude similar to fossil crude oil. While the first option is the mainstream technology to date, the second option is gaining momentum since it has certain advantages. For example, HTL can use all the biomass as feedstock, regardless of the lipid content, and can directly process wet feedstock without an energy-intensive drying process.

Another issue, which needs to be raised here, is more fundamental: just how much oil is there in a diatom? On the surface, this seems to be an easy question; we look for lipids and all forms of saturated and unsaturated fatty acids, extract them, and then measure how much are present [37]. But is this the best approach to maximize bioproducts? It is well known that by selecting certain species and manipulating nutrients in the growth media, we can affect the oil content of the microalga that we produce. Under nitrogen-starved conditions, it has also been reported that triacylglycerols in *Chaetoceros gracilis* can account for 70% of the total volume of the cell [10] (though measurement on a per weight basis would be more useful). But this reveals another potential problem with existing algal biofuel production processes. Algae typically need to be nutrient starved in order to produce high lipid content, but this can only occur at the expense of a longer growing period. This affects the overall yield of the production process on a per unit area basis over time. Algae not subjected to stress can grow rapidly, but the lipid content is limited. Furthermore, there are a wide variety of lipids found in diatoms, including membrane-associated glycolipids and extra-chloroplastic phospholipids. The proportion of these lipids can vary considerably even within a species, and it can depend on the culture conditions [38] as well as the cultivation method [39]. Thus, there is a cost for maximizing lipid content in diatoms (and other microalgae), and this involves a considerable amount of additional culture time to increase the lipid content under stress conditions. Alternatively we could be growing more total diatom biomass during the time required stressing the diatoms to increase the lipid content (where little if any additional total biomass is produced). What if we wish to produce biocrude in a way that we do not have to worry about preserving the chemical structure of the natural lipids? If the HTL process is used to obtain biocrude from the diatom, then the growth cycle and the requirement for a water-diatom separation process can be minimized. Foam fractionation might be sufficient to harvest the diatoms, because the hydrothermal process is quite tolerant to the presence of water and, in fact, requires a small amount of water. To examine the issue further, let us assume that future advances in HTL processing would eventually allow us to take advantage of all the organic carbon in the algae despite having to destroy the lipid structure. This provides another view on how to maximize useful product production from diatoms. Clearly, if our goal is to produce biocrude, then it is the organic carbon content of the diatom that we should be maximized, and not necessarily high oil or lipid content (although lipid content can affect the quality of the biocrude, at least in algae [40]), because HTL can utilize carbon in all types of biomass, including carbohydrates and proteins. The caveat is that biocrude would have to be upgraded to a fuel-grade product (this could be done in an oil refinery, but requires H_2_ input), whereas bio-oil (upgraded lipid) requires only limited treatment before it can be used as a fuel.

In light of the above, have we been mistakenly over concerned in the past about oil content in the search for an alga that would lead us to competitive biocrude production? What should be clear is the real importance of finding the sweet spot between maximizing organic carbon production and minimizing the conversion costs. This means that growth rate improvements and organic carbon should outweigh oil content in the selection of an algal (or in our case diatom) species, assuming successful HTL processing for biocrude production. This has serious implications for strain selection and genetic engineering work in all microalgae and bio-oil production manipulation.

### Breaking the cell wall

Traditionally, the approach for producing lipids from algae or diatoms and converting the it to bio-oil (or biodiesel as commonly termed by industry) has involved (a) harvesting the cells after a period of growth and application of a stress condition to maximize lipid production, (b) breaking the cells to extract the lipid, and then (c) converting the lipid to biodiesel by transesterification. While breaking open-diatom cells would be simpler (we have observed in Shenzhen that simple mechanical fracturing is effective) than other microalgae, eliminating this procedure would also be a cost benefit to any algal biofuel production process. This will be discussed below in the section on "Hydrothermal liquefaction".

Rossignol et al. [41] reported that a rapid transfer of diatoms from a region of high pressure (30–270 MPa) to one of low pressure (0.1 MPa) can cause cell breakage. Their experiments have shown that significant breakage of *Haslea ostrearia* cells occurs at 30 MPa. Kelemen and Sharpe [42] determined and compared the pressures required to disrupt 50% of various microbial cell populations. They showed that cells did not break open at any defined pressure, but rather a critical pressure had to be applied first before cell disruption could begin. The microalga, *Chlorella*, was also examined and was damaged at 48 MPa. The high pressure quick release (HPQR) cell disruption method has proven effective for recovering intracellular metabolites from diatoms. The technique is complementary to, or competitive with, conventional laboratory-scale techniques, such as sonication or shear-based systems previously use for marennine extraction. Marennine is a blue pigment produced naturally by *Haslea ostrearia* *Simonsen*, a marine diatom [43].

The HPQR technique is clearly suited for the separation of natural compounds from microalgae or cyanobacteria (e.g., nucleic acids, enzymes, proteins, and pigments). A precise particle size analysis of the resulting debris (by laser techniques) can give useful information for the choice of further metabolite separation and partial purification steps such as ultrafiltration and nanofiltration [38]. Investigations and additional engineering of existing techniques such as HPQR, and the development of other approaches for lipid release may further enhance commercial viability in the future. However, since all the research efforts thus far have been limited to laboratory exploratory experiments, the cost-effectiveness of such techniques remain unclear.

Another disruption technique is to apply one, or more, strong but extremely short electric field pulses to diatoms suspended in an aqueous medium with moderate electrical conductivity. If the field is powerful enough, a large number of transient aqueous pores should be created. Furthermore, if the internal space within diatoms has a high concentration of dissolved ions and molecules, an osmotic pressure difference will exist. Water from the external medium should move into the cells, thus increasing the pressure within the lipid-bilayer, membrane-protected regions of the diatom. The pressure difference should be large enough to rupture the diatoms. The mean lifetime of the pores is controversial, maybe one or more seconds, so more than one pulse may be needed. The temperature rise is generally small [44–47].

Richard Nuccitelli of BioElectroMed Corp. [48] told us that he had routinely used pulsed electric fields (PEF; 30 kV/cm), which make nanopores in lipid membranes and also send shock waves through water, to brake cells open. However, he did not see anything released when he tried the technique on diatoms that we supplied him. The nanosecond, pulsed electric field (nsPEF)-induced pores were very small (1 nm) and only stayed open for a few minutes at most. Thus based upon our limited experience, we conclude that this cell disruption technique does not work with the *Chaetoceros* sp. diatoms tested, but might be assessed for use with other diatoms.

In our experience, both centrifugation and freezing will break some of the diatom frustules apart, and we can extract the lipids using solvents. However, no accurate data are available on the separation of frustules for biotechnological uses.

The next section as mentioned above represents a different option for diatom biofuel production, which would entirely eliminate the requirement for cell breakage, and may well be a better approach for lowering the cost of a biocrude production process.

### Hydrothermal liquefaction of diatoms

Analogous to other microalgal species (when harvested), wet diatom biomass contains 80–90% water. Because of this high water content, and their relatively low heat capacity, diatoms and other microalgae need to be pre-treated before they can be used in heat, power generation, or other applications [49–52].

Hydrothermal liquefaction (HTL) technology operates under sub-critical water conditions and is therefore well suited to convert wet biomass, such as microalgae and perhaps diatoms (see below), into liquid fuel [53–71]. The technology is similar to the natural geological processes that led to the formation of crude oil, but occurs in minutes instead of millions of years. HTL uses water as the carrier under sub-critical conditions to decompose biomass and form more valuable, shorter chain molecules. Goudriaan et al. [72] claim that the biomass thermal processing efficiency (defined as the combined heating values of the biocrude products divided by the sum of the feedstock heating value and the external heat input) during hydrothermal treatment (HTU^®^) in a 10-kg (dry weight) h^−1^ pilot plant facility can be as high as 75%. Biocrude is the main product of the process, accounting for about 45% of the feedstock on an ash-free, dry weight basis. The biocrude had a higher heating value of 30–35 MJ kg^−1^, which might be further upgraded, if desired. Bohlmann et al. [73] developed an analogous process using a novel high-pressure, microwave reactor in order to minimize the energy consumption of algal biofuel production.

Using hydrothermal processing from 200 to 500 °C and a batch holding time of 60 min, Brown et al. [74] described the conversion of the marine microalga, *Nannochloropsis* sp., into biocrude plus a gaseous product. They estimated a higher heating value of the biocrude (about 39 MJ kg^−1^), close to that of petroleum crude. In their work, the H/C and O/C ratios for their biocrude decreased, respectively, from 1.73 and 0.12 for the products at 200 °C to 1.04 and 0.05 for the products at 500 °C. Phenol and its alkylated derivatives, derivatives of phytol and cholesterol, long-chain fatty acids, heterocyclic N-containing compounds, and alkanes and alkenes encompassed the major biocrude constituents. The two most abundant gas products were always CO_2_ and H_2_. The presence of gas-forming reactions (excluding steam reforming) was suggested by the activation energies for gas formation. Up to 90% of the chemical energy and nearly 80% of the carbon (not including CO_2_) originally present in the organisms can be recovered, either as biocrude or gas products [75].

The direct treatment of wet biomass by HTL avoids the necessity of drying, which should significantly improve the efficiency of the overall thermal process. The adaptation of a combined heat and power (CHP) plant or an internal heat exchanger network would help implement the direct processing of wet biomass. In contrast to hydrothermal and direct liquefaction technologies involving biomass, only limited studies can be found using algal biomass as a feedstock for a HTL process. Minowa et al. [76] reported a 37% oil yield (based on carbon content) from *Dunaliella tertiolecta* (moisture content, 78.4 wt%), using direct HTL at around 300 °C and 10 MPa. The biocrude obtained with a 60-min holding time and a reaction temperature of 340 °C had a (lower) heating value of 36 MJ kg^−1^ and a viscosity of 150–330 mPa. Both values are comparable to those found in fuel oil. A similar study on *Botryococcus braunii* showed a maximum yield of biocrude production at about 64% (dry wt.), obtained by HTL at 300 °C and using sodium carbonate as the catalyst [77]. Aresta et al. [77, 78] compared different conversion techniques, including pyrolysis, supercritical CO_2_, hydrothermal, and organic solvent extraction, for microalgal biodiesel production. HTL was more efficient for microalgal biocrude production than supercritical CO_2_ extraction [78]. Zhou et al. reported using HTL to convert *E. prolifera* to biocrude with a reaction time of 30 min at 300 °C when 5% Na_2_CO_3_ by weight was added [79]. Similarly, Anastasakisa and Ross [80] reported more recently on *Laminaria saccharina* (a brown macro-alga) HTL.

By converting the fatty acids in biocrude into alkanes, one could reduce the oxygen content of the bioproducts and hence improve their fuel properties. Past results indicated that biocrude produced without the help of catalysts tends to be a dark brown, foul odor, highly viscous liquid. In an inert environment, Levine et al. [81] reported that all their tested liquefaction catalysts produced greater yields of biocrude in *Nannochloropsis* sp., but the heating value of the biocrude (ca. 38 MJ/kg) as well as its elemental composition for the most part were not sensitive to the catalyst used. However, in the case of a supported Ni catalyst, the biocrude that was produced had a sulfur content that was undetectable. The desulfurization activity was unique to the Ni catalyst. Levine et al. further reported that higher amounts of protein and lipid in the algal biomass increased the yield of the biocrude and that the nitrogen content of the biocrude depended on the nitrogen content in the feedstock.

These studies suggest that HTL might be an effective option for the production of biocrude from microalgae. Nevertheless, more research in the area will be required.

Since there is no literature on the HTL of diatoms, we set out to explore this option. Using freeze-dried diatoms produced by the Shenzhen Jawkai Bioengineering R&D Center, Zhang’s group at the University of Illinois obtained the information documented in Table 1 on the constituents of Chaetoceros biomass prior to HTL. Table 2 shows some of the components identified in the biocrude produced by HTL of diatoms (*Chaetoceros* sp.). Note that we mentioned earlier that the yield of total biocrude was on the order of 36,000 l ha^−1^.

**Table 1:** Characteristics of diatom feedstock

Properties	Quantities (%)
*General properties*	
Moisture content^a^	11.45
Ash content	32.8
*Chemical composition*	
Crude protein	38.7
Crude fat	3.70
Acid detergent fiber	17.2
Neutral detergent fiber	21.0
Lignin	6.53
*Elemental composition*	
C	31.9
H	4.26
N	5.96
Si	6.76
Miscellaneous^b^	9.02
O^c^	42.1

All data calculated based on dry weight except as noted otherwise

^a^Calculated as received

^b^Sum of S, P, Mg, Ca, and Na

^c^Calculated by difference

**Table 2:** Abundant compounds found in the Biocrude by GC/MS analysis

Compounds	RT^a^
Heptane, 2,2,4-trimethyl-	8.3661
Octane, 3,3-dimethyl-	8.92
Octane, 2,2,6-trimethyl-	10.8776
Heptane, 2,2,3,5-tetramethyl-	11.2777
Octane, 2,3,3-trimethyl-	11.8334
Heptane, 2,2,4,6,6-pentamethyl-	12.8755
Octane, 2,3,6,7-tetramethyl-	13.1935
Decane, 2,6,8-trimethyl-	13.3311
Heptane, 5-ethyl-2,2,3-trimethyl-	13.7936
Dodecane, 2,7,10-trimethyl-	14.0894
Pentane, 3,3-dimethyl-	14.4501
Sulfurous acid, 2-ethylhexyl nonyl ester	17.0694
Tetradecanoic acid	31.4294
Pentadecanoic acid, methyl ester	32.398
Hexadecenoic acid, Z-11-	34.907
n-Hexadecanoic acid	35.1558
Tetradecanamide	35.225
*N*-Methyldodecanamide	35.8491
*N*,*N*-Dimethyldodecanamide	36.6065
9-Octadecenamide, (Z)-	38.5077
Hexadecanamide	38.7847
*N*-Methylhexadecanamide	39.36
*N*,*N*-Dimethypalmitamide	40.049
*N*-Decanoylmorpholine	43.8224

“Abundant” means a relative area/total identified area >2%

^a^Retention time in minutes

More specifically for Table 1, the general properties, chemical composition, and partial elemental composition were tested by the Midwest Lab (Omaha, NE). The ash content was the residue of combustion at 600 °C. Crude protein and fat were measured by the Kjeldahl method and Soxhlet extraction, respectively. Other properties were analyzed by methods suggested by the Association of Official Analytical Chemists (AOAC). CHN and silicon compositions were tested by the Microanalysis Lab at University of Illinois (Urbana, IL), using a CHN analyzer (Model CE-440, Exeter Analytical Inc., North Chelmsford, MA), and ICP, respectively.

The data in Table 2 are the organic compounds that were detected in the biocrude after HTL by the Zhang group. They found that in diatom biocrude, N-containing, hetero-aromatic compounds tended to increase with increasing reaction temperature and represented about 10% of the total identified peak area. With other microalgae, such as Spirulina, the number is >30%. This group of compounds may be viewed as undesirable products as they are chemically stable (because of their aromatic structure), and they could be hazardous to metal catalysts when further upgrading of biocrude is desired. Because denitrogenation is a major task in upgrading algal biocrude into transportation fuel, it is noteworthy that the biocrude derived from diatoms should be more readily upgradeable than biocrude from other microalgae.

Nevertheless, although HTL can substantially increase biocrude production from diatoms and other microalgae, the biocrude is of low quality compared to natural crude in terms of nitrogen and oxygen content. The nitrogen content in such biocrude is typically greater than 4%, which is substantially higher than in natural crude. Thus, catalytic upgrading will be necessary for transportation fuel as well as bioproduct production, and this is certainly a much needed area for future research.

### It is all about carbon!

If we are to produce biofuel from diatoms or other microalgae grown in outdoor ponds, it is likely that HTL in some form will be used to obtain a biocrude. Note that we said “obtain” and not “extract,” because HTL processes are all about converting carbon to oil and not about extracting existing oil from the microalgae. Note again that microalgae grow fast but produce oil slowly; we should recognize that when we grow algae for biocrude, we are actually interested in growing fixed carbon and not oil per se. The whole process should be about generating organic carbon from inorganic carbon. Whether the organic carbon is in the form of oil or sugar or anything else, HTL can generate the products that we need.

## Conclusions, current technology, and possible future developments

All the key technologies for establishing an open-pond, diatom biocrude production system are now available, even though better alternatives can and will be found as we continue to improve the process in the future.

The production data obtained in Fig. 1 were obtained using a group of small, 0.5 m diameter by 1-m high, 180 L Plexiglas tanks with open tops. In 2011, a set of four 12 m diameter by 0.5 m high, open-pond tanks were constructed and placed outdoors as a preliminary, scale-up test. These tanks were filled to a height of 0.3 m. The tanks were made of 0.6 mm, double-layered PVC material that cost less than $20 per square meter. Using these new, scaled-up outdoor mini-ponds, the average annual diatom yield was measured at 120 MT ha^−1^ (dry wt.), which compares well with the data in Fig. 1. Improved HTL processes can then be used efficiently to obtain about 40% biocrude on a dry wt. basis (up from about 33% that we currently observe) from the biomass produced. This means that production of, for example, 1 million metric tons of biocrude annually (about half a day of US petroleum usage) will require roughly 500 km^2^ of actual diatom pond surface area, and this would allow for the production of large quantities of biomass within a reasonable land area.

The production of diatoms under sub-tropical conditions is much more affected by variations in available solar irradiation than variations in temperature, as long as the temperature stays below 36 °C and above 25 °C. Our review of the literature shows that there are low temperature diatoms available, both fresh and salt water species. Therefore, they can be produced in desert areas, where saline water is available, and in the temperate zone at low temperatures. Diatoms, by the way, have been found in the desert and high altitude lakes [82].

Competitive growth of diatoms for fuel production will require a complex integrated system, if an economically viable product is desired [83]. The major costs of algae production, not counting facility investments, are fertilizers (including silicate), carbon dioxide, and electricity. From our experience, these items alone would make producing competitively priced diatom biocrude (without co-products) nearly impossible. Thus, they must be reduced or eliminated. Better yet, wherever possible, producers of diatoms should seek to be compensated for the consumption of pollutants, such as industrial heavy metal wastewater, city wastewater, and carbon dioxide. As mentioned before, the frustules from diatoms, which survive the HTL process, are potentially excellent material for the adsorption of heavy metals from industrial wastewaters. Furthermore, they might be used as environmentally friendly fillers for polymers, so widely used in our everyday activities [84, 85], or perhaps even as materials for composites [86] or metal additive manufacturing [87].

One of the largest generators of pollution in the world today is cities. Fortunately, the large amount of wastewater they produce is a very good fertilizer. Modern societies are used to paying for wastewater management. If we are to use untreated or even treated wastewater to replace fertilizers, we can save the cost of the fertilizer, and by cleaning up the wastewater, we should also gain a new revenue stream to offset the cost of the biocrude. In China lakes and rivers, polluted by wastewater discharge is a large problem. A prime example, the world renowned Dianchi Lake (in Kunming, China), is so polluted that in the summer the smell is insufferable. The reason for the pollution is that the amount of nutrient going into the lake annually exceeds what the lake itself can remediate. Billions of Chinese RMB have been devoted to solving the problem without success. The solution seems to be simple: reduce the nutrient load of the incoming water sufficiently, and in time, the lake will purify itself. In other words, further treating city wastewater at the point where it leaves the treatment plant could be accomplished using it to grow diatoms. Thus, removing nutrients and pollutants from the wastewater at the source should be a viable strategy. This is necessary because even a modern waste treatment plant removes little inorganic nutrient from city wastewater. It mostly just converts organic nutrients into inorganic ones, so clean-up is a pivotal niche for diatoms. From a technical perspective, we have demonstrated in our Shenzhen facility that we can consume the nutrients in wastewater using them to grow diatoms. If production efficiency, such as yield per unit area per unit time, is not a serious concern, and if we can match the composition of the nutrients in the treatment ponds to that required by the diatoms, we can reduce the nutrient load in the wastewater to close to zero. Nutrient Pollution, as the US Environmental Protection Agency calls it, is a serious problem in the USA. For a general description of the problem, visit https://www.epa.gov/nutrientpollution/sources-and-solutions.

From the beginning of the US Department of Energy’s Aquatic Species Program [24], funds and efforts were directed at finding the best species for the production of biofuel. Millions of dollars were spent on the effort, and thousands of species were examined in the laboratory and tested in the field. It was an effort that produced a lot of very useful scientific information, and a number of strains are still being investigated. However, our experience in Shenzhen, China (which enjoys a sub-tropical climate), producing diatoms year round and successfully competing with invading wild species required cultivating different species of diatoms at different times of the year. To meet this requirement, we developed a procedure that takes advantage of the rapid growing capability of the diatoms to continuously generate seed cultures to replace the previous production seed culture with the most competitive diatom(s) from the surrounding ocean bay; this approach has maintained the competitive vigor of the production facility continuously for almost 5 years (Fig. 1). It is well known that diatoms get smaller as they subdivide asexually (http://ag.arizona.edu/azaqua/algaeclass/lecturenotes/Diatomnotes), and new seed cultures are needed to maintain vigorous growth. Partial but continuous replacement of the indigenous diatom species under cultivation with the most competitive diatoms from the wild ensures continuous, efficient production [31]. Using locally isolated strains, we also avoid the problems that can come from introducing foreign (invasive) species into local waters.

To reduce the cost of electricity (one of the cost issues mentioned above), renewable energy such as solar or wind power could be used where appropriate. Although there are issues, such as variations in the availability of solar and wind resources and land resource availability, an open, diatom pond system could be designed to tolerate extreme, sudden, and frequent variations in power supply. Furthermore, an open, pond system can make full use of the vacant space available within the area of a conventional wind farm (or a future off-shore farm, if an ocean system can be developed). The ability to complement each other in energy use, space allocation, and the cost of land preparation, might improve the economic viability of a joint operation to insure profitability for both.

The economic viability of a diatom-for-fuel facility will require that as many co-products are produced as possible, [83] as is the case with current petrochemical refining facilities. Many potential co-products (note that the practicality of some of these might depend on the source of the water used to produce the diatoms) are possible, including specific organics like food-grade beta-carotene, pharmaceuticals, and pigments, as well as compounds like polysaccharides, carbohydrates, surfactants, and other polymers.

Oil (lipids) from diatoms can also be used to produce cooking oil in applications where HTL is not used. The high quality of the lipids produced will result in excellent oils for human consumption, and broad future availability should help with the shortage of cooking oil, which is a serious of current problem in the developing world (http://www.ocala.com/news/20080120/the-rising-price-of-cooking-oil-causes-global-food-crisis). As the world population increases, especially in the rapidly emerging economies, the demand for cooking oil will increase faster (perhaps not on an absolute basis, but certainly on a percentage basis) than the demand for fuel oil. There are other energy fuel sources, but unfortunately, there is no substitute for cooking oils from biological sources.

Note that we have not included cost analyses for the commercial production of diatoms or the products/co-products that might be produced from diatoms in this review. To our knowledge, no cost analyses for diatoms have been reported in the literature. Nevertheless, we did want to provide the reader with some information that is at least available for other microalgae in hopes that this might be helpful. For example, there are several recent cost analyses, including those for (a) a 100-ha algal biofuels facility [88]; (b) algal techno-economic, life cycle, and resource assessment (a modeling study) [89]; and (c) biomass pretreatment [90]. Furthermore, there are recent NREL reports on the economics of algal biomass production [91] and the conversion of algal biomass to biofuels [92].

## Final thoughts

Voosen [93], an E&E reporter, in an article published on March 29, 2011, said that “…During World War II, German scientists first attempted to produce oil from the microbes, discovering that green algae, when deprived of nutrients, devoted more than two-thirds of their weight to oils. The oil built up slowly, though, and the algae, investing their energy in survival, grew at a tepid rate.” This problem is still an issue today.

At MIT after WWII, mass cultivation of algae started in several large translucent bags, but protein production for food rather than oil was the focus. Voosen (in 2011) also quoted Rene Wijffels, a Wageningen University (Netherlands) engineer as remarking, “The 1950s soon saw a (research) bubble similar to the past few years, with companies saying they could rapidly expand algae production in three years with drastic drops in price [93].” These claims were quite unrealistic.

As a result, Voosen felt that the US Department of Energy’s Aquatic Species Program on lipid-producing algae, which began in 1978 in response to the oil crises of the 1970s, was justified. NREL researchers supported by the Program identified several strains out of about 3000 collected as having potential for biodiesel production. Algal test ponds were built in Roswell, N.M., to determine if the algae could be “farmed” at large scale. However, the Roswell staff quickly discovered that indigenous algae rapidly out-competed the test strains in the outdoor ponds. The Aquatic Species Program ended in 1996 as the result of rather low oil prices at the time, but the widely circulated final Program report [24] predicted “algae’s eventual return….”, a prophesy that has already come true at the research level. The preoccupation with oil content at the time in hindsight was probably not the best strategy for developing an algae-to-biofuel system since (a) algae grow fast but produce oil slowly and (b) algal farming fails primarily because wild algae rapidly displace the algae being cultivated. To be successful, the microalgal growth model should be more like agriculture. Like in farming, the first thing that needs to be done is to improve algal productivity and then protect the organisms from wild predators. Furthermore, the algal oil must be harvested efficiently and appropriate co-products produced.

The most persistent myth about algae in our opinion is an outcome of oil-yield comparisons with those of common commercial crops. One version, published in 2007 by Chisti [94], a prominent New Zealand biochemist portrayed algae as a 130 times more productive oil producer than soybeans. Since then no other algal biofuel article has been cited as widely.

“Unfortunately, people outside the algae business extrapolated speedy growth rates in open-water ponds and under ideal conditions to the industrial setting necessary for commercial cultivation,” said Greg Stephanopoulos, a biochemical engineer at MIT and a longtime expert in bacterial manipulation as conveyed by Voosen [93]. However, a frank assessment of the future of algal biofuel ponds in a 2010 Lawrence Berkeley National Laboratory report [95] found that wastewater treatment was apparently the only economically tenable route for using algae ponds, although oil might be produced concurrently. Without multiple use approaches, biocrude costs from ponds would probably be between $240 and $330 a barrel, which is 5.0–6.5 times (May 2016) the current price of fossil crude.

Our experience in China has indicated that technical problem solutions, such as controlling wild algae and predator aquatic animals, are now within reach. We have suggested directions, which may make producing biocrude cost-effective in the future, namely, by integrating diatom production with wastewater treatment, using CO_2_ from chemical or power plants, taking advantage of wind and solar power, and most importantly co-producing an array of valuable products and biofuels. It is now time to take another look at these age old problems and try once again to develop new technology that can be used at an appropriate time to ramp up a new industry with real hope for success.

## Abbreviations

CHP: Combined heat and power
HTL: Hydrothermal liquefaction
HTU^®^: Hydrothermal upgrade
HPQR: High pressure quick release
NREL: National Renewable Energy Laboratory
nsPEF: Nanosecond, pulsed electric fields
